# Supplementary material for: Effects of fragrance compounds on growth of the silkworm Bombyx mori
Source: PeerJ. 2021 Jun 16;9:e11620. doi: 10.7717/peerj.11620 (PMC8214392; doi:10.7717/peerj.11620)
Supplement: Supplemental Information 2 [file peerj-09-11620-s002.doc]

**SUPPORTING MATERIALS**

**Table S1:** The Structures of fragrance compounds in this study

| Name | Structure | CAS No. | Concentrations for toxicity study (mM) |
| --- | --- | --- | --- |
| Phantolide | 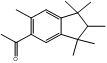 | 15323-35-0 | 1 |
| Musk AHMT | 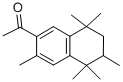 | 1506-02-1 | 1 |
| Cashmeran | 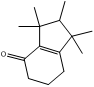 | 33704-61-9 | 1 |
| Celestolide | 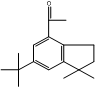 | 13171-00-1 | 1 |
| Musk tibeten |  | 145-39-1 | 0.001, 0.01, 0.1 and 1 |
| Musk xylene |  | 81-15-2 | 0.001, 0.01, 0.1 and 1 |
| Musk ketone | 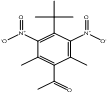 | 81-14-1 | 1 |
| Vanillin | 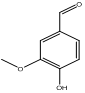 | 121-33-5 | 1 |
| Ethyl vanillin |  | 121-32-4 | 0.001, 0.01, 0.1 and 1 |
| 2-Methoxynaphthalene | 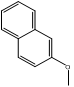 | 93-04-9 | 1 |
| Coumarin | 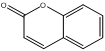 | 91-64-5 | 1 |
| Thymol | 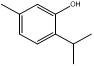 | 89-83-8 | 1 |
| Indole | 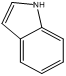 | 120-72-9 | 1 |
| Phenylacetaldehyde dimethyl acetal | 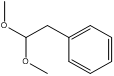 | 101-48-4 | 1 |
| 4’-Methylacetophenone | 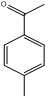 | 122-00-9 | 1 |
| 2,3,5-Trimethylpyrazine | 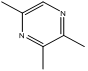 | 14667-55-1 | 1 |
| (-)-Ambroxide | 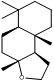 | 6790-58-5 | 1 |
| (2*E*)-2-Methyl-2-pentenoic acid | 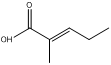 | 16957-70-3 | 1 |
| Maltol | 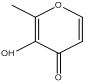 | 118-71-8 | 0.001, 0.01, 0.1 and 1 |
| Eugenol | 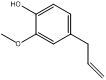 | 97-53-0 | 1 |
| Phenethyl alcohol | 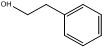 | 60-12-8 | 1 |
| Rose oxide | 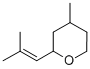 | 16409-43-1 | 1 |
| Benzyl acetate | 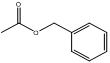 | 140-11-4 | 1 |
| α-Amylcinnamaldehyde | 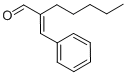 | 122-40-7 | 0.001, 0.01, 0.1 and 1 |
| Isobutyl salicylate | 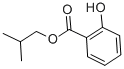 | 87-19-4 | 1 |
| α-Ionone | 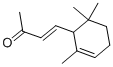 | 127-41-3 | 1 |
| Lily aldehyde | 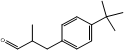 | 80-54-6 | 1 |
| 3-(4-Isopropylphenyl) Isobutyraldehyde | 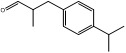 | 103-95-7 | 1 |
| α-Hexylcinnamaldehyde | 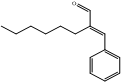 | 101-86-0 | 1 |
| Methyl 2-(Methylamino) benzoate | 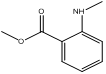 | 85-91-6 | 1 |
| 3,7-Dimethyl-2,6-octadienenitrile | 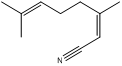 | 5146-66-7 | 1 |
| Menthol | 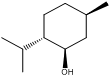 | 15356-70-4 | 0.001, 0.01, 0.1 and 1 |
| Nerolidol | 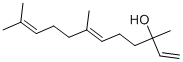 | 7212-44-4 | 0.001, 0.01, 0.1 and 1 |
| Citronellol | 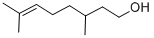 | 106-22-9 | 1 |
| Citral | 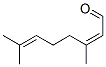 | 5392-40-5 | 1 |
| Geraniol | 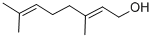 | 106-24-1 | 1 |
| (1R)-(+)-α-Pinene | 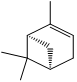 | 7785-70-8 | 1 |
| Terpinolene | 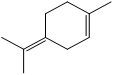 | 586-62-9 | 1 |
| Dibutyl sulfide | 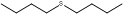 | 544-40-1 | 0.001, 0.01, 0.1 and 1 |
| Myrcene | 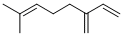 | 123-35-3 | 1 |
| Cinnamyl alcohol | 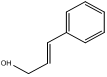 | 104-54-1 | 1 |
| Diphenyl ether | 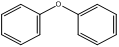 | 101-84-8 | 1 |
| (*S*)-(-)-Citronellal | **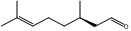** | 5949-05-3 | 1 |
| Piperitone | **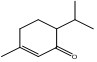** | 89-81-6 | 1 |
| *L*(-)-Borneol | 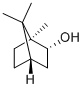 | 464-45-9 | 1 |
| Isophytol | 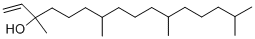 | 505-32-8 | 1 |
| 1,8-Cineole | 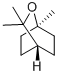 | 470-82-6 | 1 |
| Camphene | 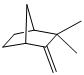 | 79-92-5 | 1 |
